# Helicobacter pylori and precancerous conditions of the stomach: the frequency of infection in a cross-sectional study of 79 consecutive patients with chronic antral gastritis in Yaoundé, Cameroon

**DOI:** 10.11604/pamj.2015.20.52.5887

**Published:** 2015-01-20

**Authors:** Firmin Ankouane, Dominique Noah Noah, Félicien Ntoné Enyime, Carole Menzy Ndjollé, Roger Nsenga Djapa, Bernadette Ngo Nonga, Oudou Njoya, Elie Claude Ndjitoyap Ndam

**Affiliations:** 1 Department of internal medicine and specialties, Faculty of medicine and biomedical sciences, University of Yaounde 1, Cameroon; 2 Department of clinical sciences, Faculty of medicine and pharmaceutical sciences, University of Douala, Cameroon; 3 Department of morphological sciences, anatomy and pathology, Faculty of medicine and biomedical sciences, University of Yaounde 1, Cameroon; 4 Department of surgery and specialties, Faculty of medicine and biomedical sciences, University of Yaounde 1, Cameroon

**Keywords:** Helicobacter pylori, gastritis, intestinal metaplasia, precancerous conditions of the stomach, Cameroon

## Abstract

**Introduction:**

The study aimed at determining the different types of precancerous conditions of the stomach and searches the frequency of *Helicobacter pylori* in these lesions in patients with chronic antral gastritis in Yaounde, Cameroon.

**Methods:**

Five gastric biopsies were performed during upper gastrointestinal endoscopy for pathology and fixed in formol 10% before being coated in paraffin. Both the modified Giemsa and Periodic acid of Shift – Alkaline blue stains were used for the histological diagnosis of *Helicobacter pylori* infection. Hematoxylyn and eosin stain was used to determine the activity of gastritis, atrophic gastritis and intestinal metaplasia in accordance to the Sydney's classification of gastritis. Data were analysed using both the Epi info 6.04 and Excel 2007 softwares. Means and their standard deviations, medians and their interquartiles (IQR) were calculated. Proportions were established for qualitative variables and chi square analysis done in this study with a p value set at 0.05.

**Results:**

Seventy-nine patients with chronic antral gastritis were enrolled, of which 43 (54.4%) were male, median age: 43 years (range from 21 to 70 years). The rate of atrophic gastritis was 74.7% (59/79). The activity of atrophic gastritis was mild in 47.5% (28/59) of cases, moderate in 47.5% (28/59) and severe in 5% (5/59). Intestinal metaplasia and follicular gastritis were present in 6.3% (5/79), and 10.1% (8/79), respectively. Concerning *Helicobacter pylori* infection, 71.2% (42/59) of patients with atrophic gastritis tested positive against 28.8% (17/59) who tested negative (p = 0.00003). Helicobacter pylori infection was related to the severity of gastric atrophy (p = 0.0001). Among patients with intestinal metaplasia and follicular gastritis, the proportion of those who tested positive for *Helicobacter pylori* infection was 80% (4/5), and 75% (6/8), respectively. There were no significant differences in the occurrence of atrophic gastritis according to age groups (p = 0.908).

**Conclusion:**

This study concludes that atrophic gastritis, which is most often caused by *Helicobacter pylori*, is the most frequent precancerous condition of stomach in Cameroon. Routine gastric sampling for pathologic analysis is mandatory for effective diagnosis and surveillance of *Helicobacter pylori* infection and precancerous conditions of the stomach.

## Introduction

Chronic gastritis, especially that associated to *Helicobacter pylori (H. pylori)* predisposes to the intestinal form of gastric carcinoma [1–3]. The histological sequence which leads to the occurrence of gastric cancer has been elucidated [1]. With regards to the pathogenesis of the intestinal types of carcinoma, it is known that atrophic gastritis is the early step, followed by intestinal metaplasia, then dysplasia before the appearance of gastric carcinoma [2–5]. Atrophic gastritis and intestinal metaplasia are thus considered early markers of gastric cancer, being precancerous conditions [6]. Pathologic analysis of gastric specimens is fundamental in diagnosing chronic gastritis, as its helps classify lesions according to the degree of severity, which vary from superficial gastritis to severe atrophic gastritis [7]. Conventional endoscopy is inadequate in establishing the extent of microscopic lesions.

*H. pylori* is highly endemic in Cameroon [8]. Prospective studies carried out using serological data have clearly established that *H. pylori* constantly leads to gastritis and is a major risk factor of both the intestinal and diffuse types of gastric cancer [9]. Furthermore, *H. pylori* has been considered by the World Health Organization (WHO) as a carcinogen, as its accounts for most gastric cancers. Its eradication will mostly be beneficial in preventing gastric cancer before the appearance of precancerous conditions [10].

However, despite the aforementioned facts, no endoscopic algorithm for the evaluation of gastritis exists in our country. Pathologic analysis of gastric specimens is not routinely practiced. Conventional endoscopy coupled to *H. pylori* detection using the rapid urease test are the only routinely practiced investigations. In the absence of pathology and chromoendoscopy, establishing the existence of precancerous conditions of the stomach in our daily practice is scarce [11].

We thus determined, through a cross-sectional study, the prevalence of atrophic gastritis, intestinal metaplasia and follicular gastritis in our environment, and also determine the frequency of H. pylori in these lesions in patient with chronic antral gastritis using the Sydney classification to evaluate gastritis.

## Methods

It was a cross-sectional study carried out from January 2013 to February 2014, both at the Yaounde Central Hospital and the University Hospital Center. Sampling was consecutive, and all patients aged between 20 and 70 years old, referred for an upper gastrointestinal (GI) endoscopy, and who had the histological diagnosis of antral gastritis were enrolled. Patients with upper GI bleeding, with active bleeding during endoscopy, those on antibiotics or antisecretory drugs within the month preceeding the endoscopy and patients with a past history of gastrectomy were excluded from the study. Five gastric biopsies were sampled during upper GI endoscopy for pathologic analysis and diagnosis of *H. pylori* infection, in accordance to the Sydney's classification of gastritis [12, 13], these biopsies included: 2 at the level of the antrum (2cm from the pylorus), 1 at the angularis and 2 in the body of the stomach. Formol 10% was used to fix the specimens before being coated with paraffin.

Standard staining techniques including hematoxylyn & Eosin, Giemsa, Periodic Acid Shift (PAS) and alkaline blue were used. Hematoxylyn & Eosin helped describe morphology, PAS and alkaline blue described lesions of malpighian intestinal metaplasia, while Giemsa was used to identify H. pylori in the bottom of gastric crypts and at the apical poles of cells. Conclusions of an upper GI endoscopy were not made known to the pathologist. The following parameters were clearly precised in the pathologist report: chronic infiltrate, atrophic gastritis (graded as absent, mild, moderate and severe), intestinal metaplasia (described as absent or present), follicular gastritis (described as absent or present), and the presence of *H. pylori* infection was ascertained when the bacterium was identified in histological sections. In this study, only the antral predominance of gastritis was taken into account.

**Ethical consideration:** the study was approved by the Ethics Committee of the Faculty of Medicine and Biomedical Sciences of University of Yaounde 1, Cameroon and signed informed consent was obtained from all included participants.

**Statistical analysis:** data was analysed using both the Epi info 6.04 and Excel 2007 softwares. For quantitative variables, means and their standard deviations, medians and their interquartiles (IQR) were calculated. Proportions were established for qualitative variables. To examine the relationship between two discrete variables, we used Pearson's? 2 test. Yates correction and Fischer's exact test were used for small sample sizes, with a p value set at 0.05

## Results

### Demographics characteristics and prevalence of precancerous conditions of the stomach

A total of 870 upper GI endoscopies were performed during the period of the study. Seventy-nine patients with chronic antral gastritis met our inclusion's criteria. The study population comprised 43 males (54.4%) and 36 females (45.6%) with a sex ratio of 1.2. The median age was 43 years old (range from 21 to 70 years). Table 1 represents the prevalence of precancerous conditions of the stomach in 79 patients with chronic antral gastritis, according to the Sydney's classification of gastritis. The prevalence of atrophic gastritis among them was 74.7% (59/79). Considering the degree of activity of gastritis, it was mild in 47.5% (28/59) of patients with atrophic gastritis, moderate in 47.5% (28/59), and severe in 5% (5/59). The prevalence of intestinal metaplasia and follicular gastritis were 6.3% (5/79), and 10.1% (8/79), respectively.

**Table 1:** Prevalence of precancerous conditions of the stomach among patients with histological proven chronic antral gastritis in Yaounde

Precancerous conditions	Number of patients	Percentage (%)
Atrophic gastritis, n (%)	59	74.7
Mild	28	47.5
Moderate	28	47.5
Severe	3	5
Intestinal metaplasia, n (%)	5	6.3
Follicular gastritis, n (%)	8	10.1

n effective % percentage

### Helicobacter pylori infection and precancerous conditions of the stomach in chronic antral gastritis

The relation between *H. pylori* infection and the occurrence of precancerous conditions of the stomach is portrayed on Table 2. As depicted by the table, 71.2% (42/59) of patients with atrophic gastritis were *H. pylori* positive against 28.8% (17/59) who were H. pylori negative (p = 0.00003). The severity of atrophic gastritis was related to the presence of H. pylori infection (p = 0.0001). The proportions of patients who were *H. pylori* positive among patients with intestinal metaplasia and follicular gastritis were 80% (4/5), and 75% (6/8), respectively.

**Table 2:** Comparison between Helicobacter pylori positive patients and Helicobacter pylori-negative patients with precancerous conditions of the stomach among patients with histological proven antral gastritis in Yaounde

Precancerous conditions	Number	*H .pylori* (+ve)	*H. pylori* (-ve)	*p*
Atrophic gastritis, n (%)	59	42 (71.2)	17(28.8)	0.00003
Mild, n (%)	28	18(64.3)	10(35.7)	
Moderate, n (%)	28	21(75)	7(25)	
Severe, n (%)	3	3(100)	0(0	0.00010
Intestinal metaplasia, n (%)	4	4(80)	1(20)	0.384
Follicular gastritis, n (%)	8	6(75)	2(25)	0.455

+ve positive, -ve negative, *H.pylori* Helicobacter pylori, n effective, % percentage

### Prevalence of precancerous conditions of the stomach with respect to age and gender in chronic antral gastritis

The distribution of precancerous conditions of the stomach with respect to gender and age is depicted on Table 3. Age did not significantly influence the occurrence of atrophic gastritis (p = 0.908). The prevalence values of atrophic gastritis at age's ranges of 20-40 years, 41-60 years, and above 60 years were 45.8%, 33.9%, and 20.3%, respectively. Concerning the degree of severity of atrophic gastritis, mild and moderate degrees of atrophic gastritis regressed with age, while severe atrophic gastritis predominated among those aged between 41 – 60 years old accounting for 66.7% (2/3) of cases. There were no significant differences in prevalence values of atrophic gastritis between males and females (49.2%, and 50.8%, respectively; p = 0.272). The prevalence values of intestinal metaplasia at age's ranges of 20-40 years, 41-60 years, and above 60 years were 20%, 40%, and 40%, respectively. The prevalence values of intestinal metaplasia in males and females were 20%, and 80%, respectively. There were no significant differences in prevalence values of follicular gastritis according to age. It was more frequent among male patients than among female patients (62.5%, and 37.5%, respectively).

**Table 3:** Prevalence of precancerous conditions of the stomach with respect to age and gender among patients with histological proven chronic antral gastritis in Yaoundé

Precancerous conditions	Patients(n)	Age (years)			Gender	
		20-40	41-60	> 60	Female	Male
Atrophic gastritis, n (%)	59	27(45.8)	20(33.9)	12(20,3)	29(49,2)	30(50,8)
Mild, n (%)	28	14(50.0)	8(28.6)	6(21.4)	14 (50.0)	14 (50.0)
Moderate, n (%)	28	12(42.9)	10(35.7)	6(21.4)	14 (50.0)	14 (50.0)
Severe, n (%)	3	1 (33.3)	2 (66.7)	0 (0.0)	1 (33.3)	2 (66.7)
Intestinal metaplasia, n (%)	5	1 (20.0)	2 (40.0)	2(40.0)	4 (80.0)	1 (20.0)
Follicular gastritis, n (%)	8	3 (37.5)	2 (25.0)	3(37.0)	3 (37.5)	5 (62.5)

n effective, % percentage

## Discussion

In Cameroon, the incidence, prevalence and mortality of gastric cancer has not been clearly established, although gastric cancer has been reported to be the second most common gastrointestinal malignancy [14, 15]. In Europe, notably in France where data exists, gastric cancer is the fourth most common gastrointestinal cancer, with a poor prognosis, having a 5-years survival rate of about 25% [16–18]. The histological sequence leading to gastric cancer has been established, and passes through precancerous conditions [1, 4]. It successively passes through atrophic gastritis, intestinal metaplasia, dysplasia before carcinoma [1–3].

In this study, we found a high rate of atrophic gastritis (74.7%) in patients with chronic antral gastritis, but intestinal metaplasia (6.3%) and follicular gastritis (10.1%) were rare. This finding is similar to those found in low risk groups for gastric cancer. Paradoxically, it is different from the findings of a study in Cote d'Ivoire, a country whose settings are similar to ours and where, these lesions were very frequent [19]. It is known that the distribution of precancerous conditions of the stomach varies with respect to countries [17, 18, 20]. In very high risk groups like Japan and China, and certain high risk groups (blacks and Hispanics) in the United States of America, the rate of precancerous conditions of the stomach is generally very high [20–23]. Discrepancies in the diagnosis of precancerous conditions of the stomach may also be related to the site of gastric biopsy. The angularis, for instance, habours more precancerous conditions than the body and the antrum [24]. In this study, analysis was limited to the antrum this may explain ours results and may limit the value of our findings. Also, the relatively small sample of this study constitutes a limit.

*H. pylori* infection is responsible for 80% of atrophic gastritis and is related to the development of precancerous conditions of the stomach and their progression to carcinoma [3, 5, 9, 19, 24]. The association between *H. pylori* infection and atrophic gastritis or intestinal metaplasia increases the risk of gastric cancer to five - six-fold [3, 6, 18, 25]. *H. pylori* infection is both related to the severity of histological lesions and the activity of chronic antral gastritis [3, 9, 25, 26]. Results of this study indicated that *H. Pylori* infection is frequent in precancerous conditions of the stomach and it's associated to the severity of histological lesions of the stomach (71.2% in atrophic gastritis and 80% in intestinal metaplasia).

In western countries, the majority of precancerous conditions of the stomach and gastric cancers are diagnosed beyond 75 years [16–18]. In this study, we found a high rate of precancerous conditions of the stomach before the age of 60 years. This early onset of the development of *H. pylori* related gastric cancer has been clearly established in many studies carried out in other developing countries [5, 8, 19, 20]. Intestinal metaplasia was more frequent among female patients (80%), though this could not be accounted for.

## Conclusion

Atrophic gastritis often caused by *H. pylori*, is the most frequent precancerous condition of the stomach in Cameroon. Routine gastric sampling for pathologic analysis is mandatory for effective diagnosis and surveillance of *H. pylori* infection and precancerous conditions of the stomach. The progression of precancerous conditions to gastric cancer which mortality remains high will be halted by the eradication of *H. Pylori* infection.
